# LLM-Enhanced Multimodal Framework for Drug–Drug Interaction Prediction

**DOI:** 10.3390/biomedicines13102355

**Published:** 2025-09-26

**Authors:** Song Im, Younhee Ko

**Affiliations:** Department of Biomedical Engineering, Hankuk University of Foreign Studies, Yongin 17035, Gyeonggi-do, Republic of Korea; song.im@hufs.ac.kr

**Keywords:** drug–drug interaction, DDI prediction, deep learning, CTET, large language model (LLM) embeddings

## Abstract

**Background:** Drug–drug interactions (DDIs) involve pharmacokinetic or pharmacodynamic changes that occur when multiple drugs are co-administered, potentially leading to reduced efficacy or adverse effects. As polypharmacy becomes more prevalent, especially among patients with chronic diseases, scalable and accurate DDI prediction has become increasingly important. Although numerous computational approaches have been proposed to predict DDIs using various modalities such as chemical structure and biological networks, the intrinsic heterogeneity of these data complicates unified modeling; **Methods:** We address this challenge with a multimodal deep learning framework that integrates three complementary, heterogeneous modalities: (i) chemical structure, (ii) BioBERT-derived semantic embeddings (a domain-specific large language model, LLM), and (iii) pharmacological mechanisms through the CTET proteins. To incorporate indirect biological pathways within the PPI network, we apply a random walk with restart (RWR) algorithm. **Results:** Across features combinations, fusing structural feature with BioBERT embedding achieved the highest classification accuracy (0.9655), highlighting the value of readily available data and the capacity of domain-specific language models to encode pharmacological semantics from unstructured texts. **Conclusions:** BioBERT embeddings were particularly informative, capturing subtle pharmacological relationships between drugs and improving prediction of potential DDIs. Beyond predictive performance, the framework is readily applicable to real-world clinical workflows, providing rapid DDI references to support the polypharmacy decision-making.

## 1. Introduction

The concurrent use of multiple medications—commonly referred to as polypharmacy—has become increasingly common, particularly among older adults and patients with multiple chronic conditions [1]. While polypharmacy is often necessary for comprehensive disease management, it also elevates the risk of drug–drug interactions (DDIs), which can lead to unexpected adverse effects or reduced therapeutic efficacy [2,3]. Identifying potential DDIs therefore remains a critical challenge in clinical pharmacology and drug development.

Traditional methods for detecting DDIs—including in vitro assays, animal studies, clinical trials, and post-marketing surveillance—continue to provide critical insights [4,5,6,7]. However, these approaches are time- and resource-intensive and infeasible for evaluating the vast number of possible drug combinations. This challenge is particularly pronounced for newly approved or investigational drugs, which often lack sufficient interaction data to support empirical assessment [8].

To address these limitations, a range of computational approaches have been proposed for DDI prediction [9,10,11,12,13]. Early methods focused on structural similarity or molecular fingerprints, based on the assumption that chemically similar drugs exhibit similar interaction profiles [14,15]. Another model also incorporated biological features, such as protein–protein interaction (PPI) networks, to infer DDIs through indirect functional associations [16]. More recently, natural language processing (NLP) techniques have been applied to extract semantic features from biomedical studies, drug labels, and curated knowledge bases [17,18]. These approaches aim to capture multi-level semantics information, including drug-specific context, to improve the accuracy of DDI prediction.

Furthermore, recent deep learning frameworks have integrated multiple modalities—structural, biological, and semantic—into unified models. Several studies have also approached DDI prediction as a multi-class classification problem, targeting fine-grained interaction types (e.g., 65 or more categories) [15,19,20,21,22,23,24]. Despite these advances, many existing models still rely on fixed input formats or curated interaction graphs, which may hinder generalization to newly approved or less-characterized compounds.

To overcome these limitations, we present a multimodal deep learning framework that combines three complementary sources of information: (i) molecular structural similarity, (ii) protein similarity derived from PPI networks, and (iii) semantic embeddings derived from BioBERT, a biomedical language model pre-trained on large-scale corpora [25]. Large language models (LLMs) have transformed biomedical research by enabling the extraction of complex semantic knowledge from unstructured texts. In particular, they have shown promising applications in drug discovery tasks, including drug–drug interaction and drug-target prediction [18,26]. As a domain-specific large language model trained on PubMed abstracts and full-text articles, BioBERT extracts pharmacologically meaningful representations from drug descriptions that are well-suited for DDI prediction. By integrating the three modalities, the model captures both chemical structure and semantic/biological contexts for each drug.

The proposed model is implemented as a flexible MLP architecture that accommodates varying input combinations. While the full framework supports all three modalities, empirical evaluation demonstrated that the combination of structural similarity and semantic embeddings—without protein-based features—yielded the best classification accuracy. This modular design enables the model to perform robustly even when certain data types are unavailable. The findings indicate that structural and semantic features alone can achieve high accuracy, while reducing dependence on complex biological inputs.

Importantly, the required inputs are broadly accessible. The semantic embeddings are derived from general drug descriptions without the need for curated DDI annotations or complex graph structures and structural features can be computed from widely available molecular representations (e.g., SMILES). In contrast, protein similarity features derived from PPI networks require more extensive biological information, which is often incomplete or unavailable, particularly for investigational compounds. Our results showed that the combination of structural and semantic features (SSP + BioBERT) outperformed the full model that also included protein-based features (PSP). This suggests that the best-performing configuration depends on readily available data, improving both scalability and applicability across a wide range of drug compounds, including newly approved or less-characterized drugs.

## 2. Materials and Methods

### 2.1. Drug–Drug Interaction Data

We obtained drug information from DrugBank (version 5.1), including chemical structure such as SMILES strings, drug names, and textual descriptions. DDI type (class) labels were obtained from DrugBank via DeepDDI [13]. Of the 86 distinct interaction types, we excluded 7 types due to insufficient data, resulting in 79 DDI types used for training and evaluation (Supplementary Table S1). In total, 1705 drugs were included in the final dataset, yielding 178,849 drug pairs used for model training and evaluation.

### 2.2. BioBERT Embedding

To extract semantic features of each drug, we used BioBERT, a pre-trained biomedical language model that produces 768-dimensional embeddings [24]. Drug names and general descriptions were obtained from DrugBank. Among the 9377 drugs with available descriptions, 9294 drugs had input within BioBERT’s maximum sequence length (e.g., 512-token) and were processed without truncation. If a drug’s name was not included in its description, we prepended the name. Otherwise, the description was used as provided. Each input was encoded once, and hidden state of the [CLS] token was taken as drug’s semantic representation, resulting in a 768-dimension BioBERT embedding per drug.

### 2.3. SSP: Structural Similarity Profile

SSP was calculated from molecular fingerprints derived from SMILES representations. To maximize coverage, we collected SMILES for 13,082 drugs from DrugBank [27], PubChem [28] and ChEMBL [29]. Each SMILES was converted into an extended-connectivity fingerprint (ECFP) using RDKit, yielding binary vectors that indicate the presence or absence of specific chemical substructures. We evaluated both ECFP4 and ECFP6 to examine the effect of fingerprint resolution. While ECFP6 generally resulted in higher average accuracy, the ECFP4-based SSP yielded the single best performance in one setting (Supplementary Table S4), suggesting that optimal fingerprint resolution may depend on the embedding combination. Overall, however, performance differences due to fingerprint resolution were minimal.

The pairwise structural similarity between drugs was computed using the Tanimoto coefficient. For each drug, we then constructed an SSP feature vector consisting of its Tanimoto similarities to all other drugs, resulting in an initial dimension of 13,082. To reduce dimensionality while preserving most of the variance, principal component analysis (PCA) was applied, and 200 components were retained for model training.

### 2.4. PSP: Protein Similarity Profile

To capture protein-level similarities between drugs, we constructed Protein Similarity Profile (PSP) features from drug–protein associations and a protein–protein interaction (PPI) network. The PPI network was compiled from STRING [30], a comprehensive resource of known protein–protein interactions that include direct (physical) and indirect (functional) associations derived from experimental, co-expression, computational inference, and curated databases.

We adopted the definition of CTET proteins from PRID [16] and collected a total of 3955 CTET proteins associated with 6747 drugs in DrugBank. These proteins serve as molecular mediators of pharmacokinetics and pharmacodynamics, and drug–drug interactions (DDIs) often arise from their functional overlap or perturbation of shared biological pathways.

Following the PSP procedure in PRID, we applied the random walk with restart (RWR) algorithm [31] to the PPI network to incorporate indirect functional connections among CTET proteins associated with different drugs. Since most drugs are linked to only a few CTET proteins and often do not share direct overlap, RWR allows consideration of indirect functional relationships by propagating signals from each drug’s seed proteins to their neighbors [32]. The resulting diffusion scores were then used to construct the PSP vector for each drug, representing the network-informed protein similarity profile. This method enables the model to reflect pharmacologically relevant similarities even when two drugs do not share CTET proteins directly by considering the topology of the underlying PPI network.

### 2.5. Model Architecture: Feature Processing and DDI Prediction

**Figure 1:**
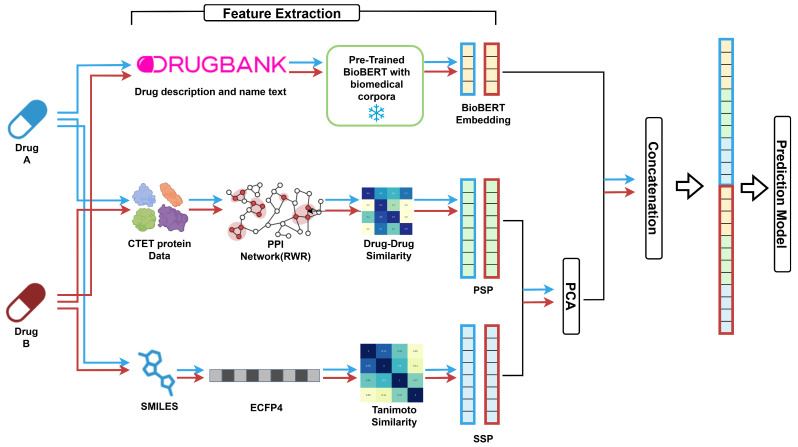
Overall framework of the proposed DDI prediction model. Features are extracted from three modalities: BioBERT embeddings (drug description and name), protein similarity profiles (PSP) computed via random walk with restart (RWR) on CTET (Carrier, Transporter, Enzyme, and Target) over PPI networks, and structure similarity profiles (SSP) based on SMILES-derived ECFP4 fingerprints with Tanimoto coefficient. In the heatmaps, more intense colors represents higher similarity between two drugs in terms of their targets and chemical structures, respectively. Extracted features are concatenated and fed into multi-layer perceptron classifiers for final prediction. The source code and data are available on GitHub (https://github.com/SongIm/ddi-prediction, accessed on 21 August 2025).

The overall framework of our proposed DDI prediction model is shown in Figure 1, and its step-by-step workflow is detailed in Supplementary Algorithm S1. We integrate three modalities to represent each drug: text-based embeddings, structural similarity features, and protein interaction-based features. For each drug pair, different combinations of SSP, PSP, and BioBERT embeddings were evaluated depending on the experimental setting. Using PCA, we reduced SSP to 200 dimensions and PSP to 300, while BioBERT embeddings were retained at their native 768 dimensions per drug.

- PCA was applied to SSP and PSP to reduce dimensionality while preserving ≥ 95% of the variance. In contrast, applying PCA to BioBERT embeddings generally degraded performance in preliminary experiments and we therefore used the original 768-dimensional vectors, suggesting that their semantic information was critical for prediction.
- After feature extraction, the selected representations were concatenated into a single feature vector (Figure 1). Before any dimensionality reduction, the input vector was passed through a projection layer—a learnable linear map with the same input and output size as the concatenated feature. The projection layer re-weights and linearly mixes feature dimensions to mitigate cross-modal scale/variance mismatches and map them into a better-aligned joint space before compression. The projection output was then fed to a hidden layer, followed by an output layer that mapped the hidden dimension to logits for the 79 DDI classes.
- The network architecture was determined using a two-stage hyperparameter optimization process. First, grid search was used to identify the number of hidden layers (1–5). We then employed Optuna [33] to tune the hidden dimension, dropout rate, and learning rate for each feature combination. The final architecture consisted of a projection layer for feature re-weighting/alignment, followed by a hidden layer for dimensionality reduction, and an output layer for classification. ReLU activation and dropout were applied after each linear transformation to improve generalization.
- After fixing the best hyperparameters, we trained from scratch for up to 300 epochs with Adam and cross-entropy loss. To ensure sufficient convergence, early stopping (patience = 20) was activated only after epoch 200, and the best validation-accuracy checkpoint was saved. Next, we resumed from this checkpoint and performed fine-tuning for up to 100 additional epochs with ReduceLROnPlateau (mode = max, factor = 0.1, patience = 5, min_lr = 1 × 10^−6^); early stopping (patience = 20) was also applied in this stage. Test metrics were reported from the best validation checkpoint of the fine-tuning stage. The overall MLP configuration is summarized in Supplementary Table S2, and detailed hyperparameters for each feature combination are provided in Supplementary Table S3. The dataset was split into training, validation, and test sets with a ratio of 0.64:0.16:0.20, using stratified sampling to preserve the distribution of all 79 interaction classes.

## 3. Results and Discussion

### 3.1. Performance Evaluation

To evaluate the contribution of each modality, we conducted an ablation study across different feature combinations (Supplementary Tables S4 and S5). Models using single modality features, such as BioBERT only or SSP only, achieved moderate accuracy with 0.9584 and 0.9495, respectively. Incorporating additional modalities into the model consistently improved performance. For example, combining SSP and BioBERT yielded the highest accuracy of 0.9655, outperforming all single-modality models.

These results highlight the complementary nature of structural similarity and semantic embedding. While protein similarity profiles (PSP) alone showed relatively lower performance (0.9292), integrating them with BioBERT or SSP features (PSP + SSP or PSP + BioBERT) improved performance relative to each single-modality baseline, demonstrating the benefit of multi-modal feature fusion for DDI prediction; however, adding PSP on top of the best two-modality setting (SSP + BioBERT) did not yield additional gains. The performance gain from the projection layer was consistent across both shallow and deeper MLP architectures, indicating its general applicability.

Additionally, introducing a projection layer consistently improved performance across most feature combinations. For example, in the SSP + BioBERT setting, the model with a projection layer achieved an accuracy of 0.9655, compared to 0.9611 without it. This suggests that the projection layer helps re-weight and align heterogeneous feature spaces before compression. Studies in multimodal and contrastive learning—such as CLIP [34] and SimCLR [35]—have demonstrated that applying a learnable projection head prior to task-specific loss functions improves alignment in shared latent spaces and enhances downstream performance.

**Figure 2:**
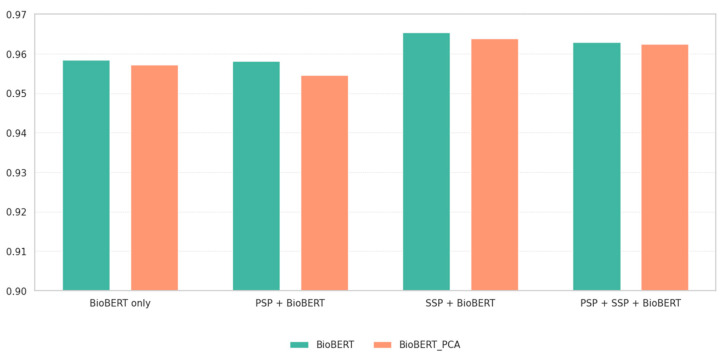
Accuracy comparison of BioBERT vs. BioBERT_PCA embeddings across feature combinations. PCA consistently reduced performance compared to using full BioBERT embeddings.

We also observed that applying PCA to BioBERT embeddings generally led to slightly reduced performance compared to using the full 768-dimensional embeddings. As shown in Figure 2, this pattern was consistent across all evaluated feature combinations, including when BioBERT was combined with SSP or PSPs. In every case, PCA-reduced embeddings led to lower prediction accuracy. This suggests that BioBERT embeddings already provide a compact yet semantically dense representation, and further compression may compromise their predictivity.

Beyond accuracy, our best-performing model (SSP + BioBERT) achieved a macro-average AUC of 0.990 and a macro-averaged AUPR of 0.9688 on the test set, confirming its strong discriminative capability across all interaction types.

**Figure 3:**
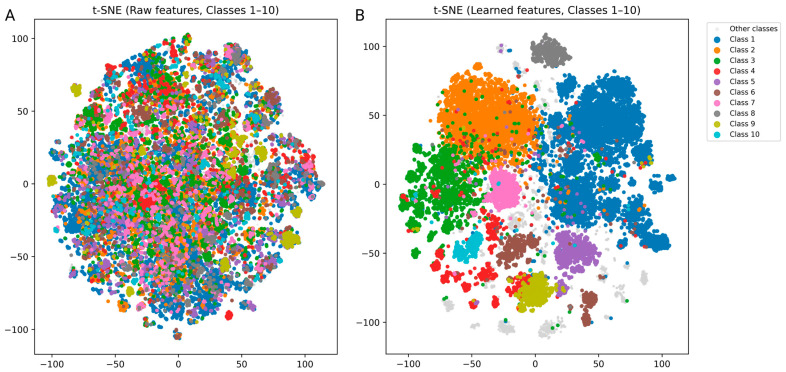
(**A**) Raw input features show no clear separation among interaction classes. (**B**) Learned representations form distinct clusters corresponding to different DDI classes, suggesting that the model captures pharmacologically meaningful structure.

To further assess whether the learned embeddings capture meaningful pharmacological information, we visualized the raw and learned features using t-SNE (Figure 3). While the raw features showed little separation across interaction classes, the learned representations exhibited clearer clustering patterns, indicating that the model encodes clinically relevant relationships between drugs.

### 3.2. Comparison with Existing Methods

**Figure 4:**
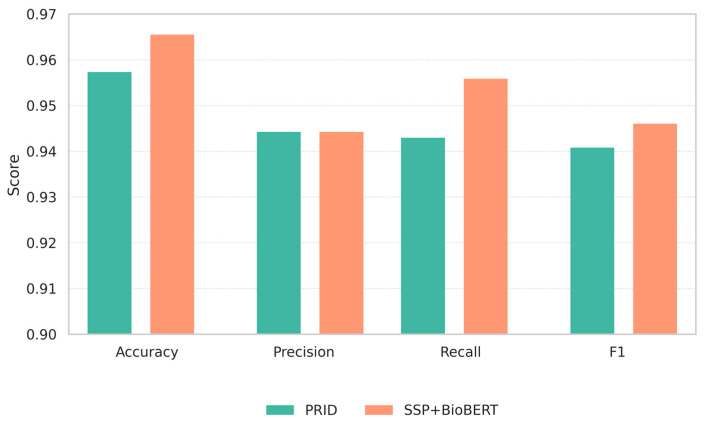
Comparison of overall accuracy and macro-averaged precision, recall and F1 between PRID and our proposed model on their respective test sets.

**Figure 5:**
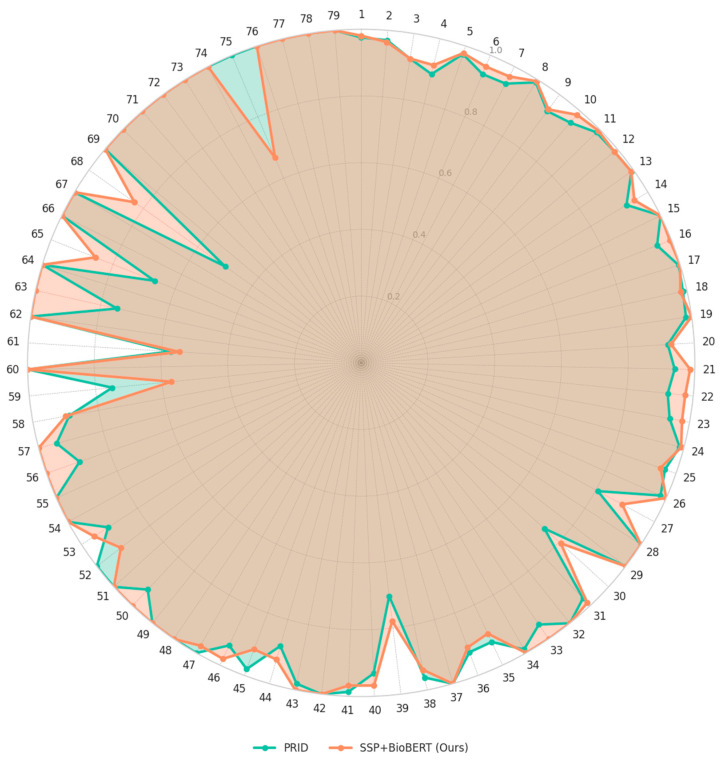
Class-wise comparison of prediction accuracy between PRID and our proposed SSP + BioBERT model. The radar plot shows that our model generally outperforms PRID across most classes.

We evaluated the class-wise prediction accuracy of our proposed model (SSP + BioBERT) and compared it with PRID, a state-of-the-art DDI prediction model. Figure 4 summarizes global performance: our model (SSP + BioBERT) shows higher accuracy and macro-F1 than PRID, with similar macro-precision and higher macro-recall. As shown in Figure 5, the radar chart illustrates the per-class performance across the 79 interaction types. Overall, our model demonstrated comparable or superior performance to PRID for most classes. Notably, our model outperformed PRID in 38 classes, while PRID performed better in 15 classes.

For example, in type 68 (“the risk of severity of hypotension can be increased when Drug A is combined with Drug B”), our model achieved substantially higher accuracy than PRID. Similar improvements were observed in type 63 (“the absorption of Drug B can be decreased when combined with Drug A”). These types tend to have relatively few positive samples in the training data, suggesting that our model generalizes better under data scarcity. We attribute this robustness to the effective fusion of structural similarity and semantic embedding, which provides more informative and complementary features than the combination of structural and protein similarity used in PRID.

### 3.3. Clinical Implication of False Positive Cases

**Figure 6:**
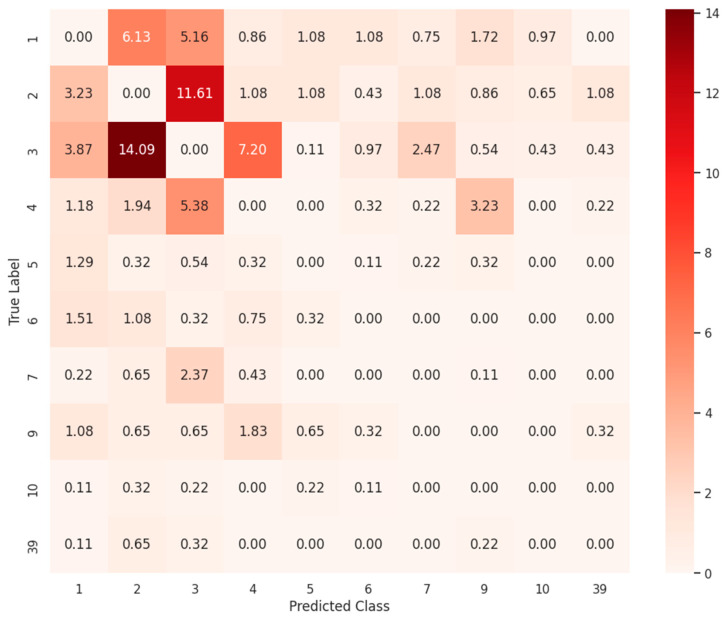
Misclassification heatmap for the top 10 most frequently misclassified DDI types. The values in the heatmap represent the normalized proportion of misclassified samples between the corresponding true and predicted interaction types, where higher values indicate greater confusion.

We further investigated the misclassification patterns of our model to identify potential limitations and areas for improvement. Figure 6 shows the heatmap of the top 10 most misclassified interaction types, revealing that interaction types 1, 2, 3 and 4 accounted for the majority of errors. DDI type 3 showed the highest misclassification ratio, accounting for 21.72% of all misclassified samples, despite comprising only 11.52% of the test set (Table 1). DDI type 2 similarly exhibited a high misclassification ratio of 21.07%, with a test sample proportion of 17.29%. DDI type 1, while the most frequent DDI type in the dataset (32.38% of test samples), accounted for 12.32% of misclassification. These three DDI types are pharmacologically related. Inhibition of drug metabolism (DDI type 2) can lead to increased serum concentration (DDI type 3), which may result in heightened toxicity (DDI type 1). This mechanistic relationship likely contributes to model confusion among these classes.

To determine whether some false positives might reflect plausible pharmacological reasoning rather than model error, we first examined cases labeled as Class 2 but predicted as Class 3. As summarized in Table 2, these pairs involve inhibition of CYP enzymes, particularly CYP3A4 affecting Drug B, which is expected to increase systemic exposure. This consistent pharmacokinetic (PK) pattern is well documented in the literature and drug labels and suggests that the model is capturing mechanistic nuances that are not explicitly reflected in the current DDI class labels. In some cases, the predicted Class 3 outcome may actually offer a more accurate pharmacological interpretation than the original Class 2 label.

**Figure 7:**
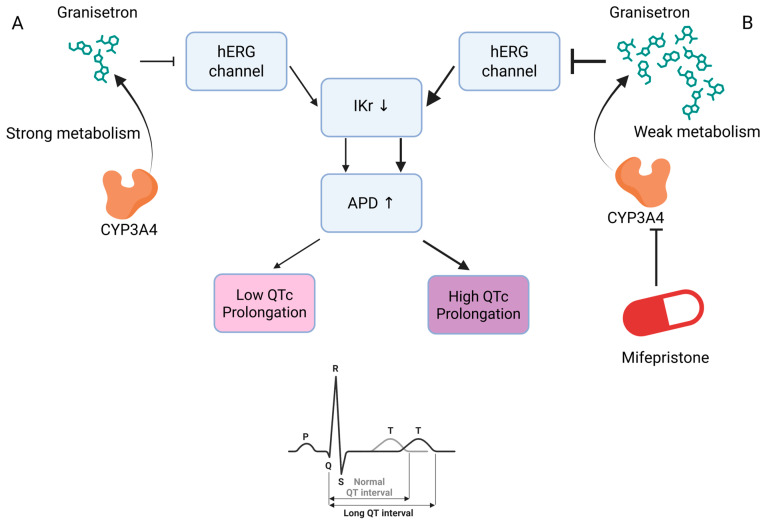
Granisetron–mifepristone pathway. (**A**) Baseline (CYP3A4 active): low granisetron exposure results in low QTc prolongation. (**B**) Granisetron + mifepristone (CYP3A4 inhibition): high granisetron exposure resulted in high QTc prolongation. ⊣ inhibition; → causal flow; IKr: rapid delayed rectifier K^+^ current; APD: action potential duration. Created in BioRender. Im, S. (2025) https://BioRender.com/i1bqtg8.

We next examined cases labeled as Class 3 but predicted as Class 7. A representative example is mifepristone and granisetron shown in Figure 7. Mifepristone inhibits CYP3A4, thereby increasing the serum concentration of granisetron (Class 3). The higher exposure produces hERG block, which reduces IKr, prolongs APD, and consequently increases the risk of QTc prolongation (Class 7). Hence, this type of misclassification follows a plausible PK → PD cascade rather than a random error; the upstream PK effect (Class 3) can manifest as a downstream PD risk (Class 7).

Third, several pairs labeled Class 9 but predicted Class 4 show the mirror-image cause–effect of the first pattern. Enzyme induction (e.g., UGT or CYP3A4/2C8) increases clearance, which lowers exposure—a mechanistically coherent path from induction (Class 9) to reduced concentration (Class 4).

Lastly, some pairs labeled Class 41 (reduced vasoconstrictor activity) but predicted as Class 5 (increased hypotensive effect) are more naturally interpreted as additive pharmacodynamic effects when two BP-lowering agents are co-administered. In such cases, the model’s Class 5 prediction more closely matches the combined PD liability than the antagonistic framing implied by Class 41.

Collectively these findings indicate that a subset of false positives arise from mechanistically consistent cascade or additive effects that fall between or across our current label definitions. This highlights both the model’s sensitivity to pharmacological cues and an opportunity to refine label granularity.

## 4. Conclusions

We presented a multimodal deep learning framework that integrates structural, semantic, and protein-based features to predict drug–drug interaction (DDI) types. By leveraging BioBERT-derived embeddings alongside chemical and biological information, our model achieved high predictive performance across 79 interaction classes. These results highlight the potential of language models to encode latent pharmacological knowledge, while also revealing limitations in capturing fine-grained mechanistic distinctions.

Several limitations of the proposed model warrant discussion. First, it appears to rely more heavily on underlying mechanisms (e.g., enzyme inhibition) than on observable pharmacological outcomes. While this tendency may benefit mechanistic interpretation, it also suggests the model may not fully capture whether a drug increases or decreases an effect, as described in drug labels.

Second, the current model lacks explicit knowledge of pharmacological pathways or ontologies. As a result, it cannot reason effectively over complex or multi-step interactions that extend beyond surface-level textual patterns. This limitation likely affects its performance in cases where drug effects depend on indirect or context-specific mechanisms.

Third, while the semantic embeddings derived from BioBERT contributed meaningfully to model performance, they are primarily based on surface-level language patterns and pharmacological descriptions, rather than mechanistic interaction evidence. As a result, two drugs with similar indications or side effect profiles may yield similar embeddings—even if their roles in interactions differ substantially. This can lead to confusion between interaction classes, especially when mechanistic distinctions are underrepresented in the textual input. In such cases, semantic similarity may not align with interaction-specific differences, highlighting a limitation of language-based representations.

Future work can proceed in several directions to further improve DDI prediction. First, while BioBERT embeddings demonstrated substantial utility in capturing semantic features, these representations were derived from a general biomedical corpus without explicit DDI supervision. Fine-tuning BioBERT or other biomedical language models (e.g., PubMedBERT [26]) on DDI-specific corpora—such as curated interaction reports, clinical notes, or pharmacological texts—may yield more informative embeddings that capture finer-grained interaction semantics. Furthermore, generative LLMs could be leveraged not only for extracting but also for generating enriched textual representations, thereby providing deeper pharmacological semantics for DDI prediction. This approach could enhance the model’s ability to distinguish mechanistic nuances and resolve ambiguities among similar DDI classes. Second, the interaction type labels in this study were based on those defined by DeepDDI [14], which reflect DrugBank at the time of its publication in 2018. Given the substantial updates to DrugBank since then, future work could benefit from re-extracting and reorganizing the interaction labels using the most recent version, potentially capturing newly discovered interactions and improving label completeness.

Overall, our findings provide a foundation for future developments through domain-specific fine-tuning and integration of updated interaction annotations.

## Figures and Tables

**Table 1:** Top 10 most misclassified DDI types ranked by misclassification rate.

Class	Description	Test Samples	Test Sample Ratio (%)	Misclassified Count	Misclassified Ratio (%)
3	The serum concentration of Drug b can be increased when it is combined with Drug a.	4122	11.52	268	21.72
2	The metabolism of Drug b can be decreased when combined with Drug a.	6186	17.29	260	21.07
1	The risk or severity of adverse effects can be increased when Drug a is combined with Drug b.	11,583	32.38	152	12.32
4	The serum concentration of Drug b can be decreased when it is combined with Drug a.	1683	4.71	128	10.37
9	The metabolism of Drug b can be increased when combined with Drug a.	935	2.61	68	5.51
7	Drug a may increase the QTc-prolonging activities of Drug b.	1180	3.3	51	4.13
6	The therapeutic efficacy of Drug b can be decreased when used in combination with Drug a.	1488	4.16	37	3
5	Drug a may increase the hypotensive activities of Drug b.	1647	4.6	36	2.92
10	Drug a may increase the anticoagulant activities of Drug b.	631	1.76	20	1.62
39	The serum concentration of the active metabolites of Drug b can be reduced when Drug b is used in combination with Drug a resulting in a loss in efficacy.	65	0.18	19	1.54

**Table 2:** Representative misclassified cases where the predicted Class may better reflect the true DDI mechanism than the true labels.

True Class	Predicted Class	Drug A	Drug B	Comment
2	3	Ketoconazole	Delavirdine	CYP3A4 inhibition by ketoconazole increases delavirdine serum levels [36].
2	3	Voriconazole	Amiodarone	CYP3A4 inhibition by voriconazole increases amiodarone serum levels [37].
2	3	Cobicistat	Ketamine	CYP3A4 inhibition by cobicistat increases oral ketamine serum levels [38].
3	7	Mifepristone	Granisetron	CYP3A4 inhibition by mifepristone may increase the risk of QTc prolongation with granisetron [39,40].
3	7	Mifepristone	Ziprasidone	CYP3A4 inhibition by mifepristone may increase the risk of QTc prolongation with ziprasidone [40,41].
3	7	Mifepristone	Iloperidone	CYP3A4 inhibition by mifepristone may increase the risk of QTc prolongation with iloperidone [41,42].
9	4	Rifampicin	Lamotrigine	UGT induction by rifampicin decreases lamotrigine serum levels [43].
9	4	Rifapentine	Dabrafenib	CYP3A4/2C8 induction by rifapentine decreases dabrafenib serum levels [44,45].
41	5	Silodosin	Lofexidine	Coadministration of the α1-blocker silodosin and the central α2-agonist lofexidine may increase the risk of hypotension through additive effects [46,47].

## Data Availability

Code and data are available at GitHub: https://github.com/SongIm/ddi-prediction (accessed on 21 August 2025).

## Supplementary Materials

The following supporting information can be downloaded at: https://www.mdpi.com/article/10.3390/biomedicines13102355/s1, Algorithm S1: Workflow of the proposed DDI prediction framework. Table S1: Full list of Drug–Drug Interaction (DDI) types and their definitions; Table S2: Summary of MLP architecture and training setting; Table S3: Hyperparameter settings for the best performing feature combinations; Table S4: Accuracy by feature combination and MLP layer configuration (ECFP4); Table S5: Accuracy by feature combination and MLP layer configuration (ECFP6).

## Abbreviations

The following abbreviations are used in this manuscript:

LLM	Large Language Model
CTET	Carrier, Transporter, Enzyme, and Target
DDI	Drug–drug Interaction
RWR	Random Walk with Restart
NLP	Natural Language Processing
PPI	Protein–protein Interaction
SMILES	Simplified Molecular Input Line Entry System
ECFP	Extended-Connectivity Fingerprint
PD	Pharmacodynamics
PK	Pharmacokinetics
