# Supplementary material for: LLM-Enhanced Multimodal Framework for Drug–Drug Interaction Prediction
Source: Biomedicines. 2025 Sep 26;13(10):2355. doi: 10.3390/biomedicines13102355 (PMC12561088; doi:10.3390/biomedicines13102355)
Supplement: Supplementary file 1 [file biomedicines-13-02355-s001.zip › biomedicines-3859126-supplementary.pdf]

Algorithm S1. Workflow of the proposed DDI prediction framework

```
Inputs:
     D: set of Drugs
     P = {(a, b, y_ab)}: labeled drug pairs with y_ab ∈ L, |L| = 79
     t(d): drug description text for drug d
     s(d): structural information for drug d
     p(d): protein related information for drug d
     Embedding functions Bio(t), SSP(s), PSP(p)
Output: Trained classifier f(·) and evaluation metrics
1.  Embedding extraction
    for each drug d ∈ D do
     e_b(d) ← Bio(t(d))           // BioBERT embedding
     e_s(d) ← SSP(s(d))           // Structural similarity profile (PCA-reduced)
     e_p(d) ← PSP(p(d))           // Protein similarity profile (PCA-reduced)
    end for
2.  Feature construction
    for each (a, b, y_ab) ∈ P do
     x_ab ← concat( e_b(a), e_b(b),
                    e_s(a), e_s(b),
                    e_p(a), e_p(b) ) // combination depends on experimental setting
     assign label y_ab
    end for
3.  Data split
     (X_train, X_val, X_test,
      y_train, y_val, y_test) ← StratifiedSplit(X, y, ratio=0.64:0.16:0.20)
4.  Model training
    Initialize MLP with:
     - Projection layer Proj(x) = W_proj x + b_proj
     - Hidden layer h = ReLU(Dropout(W_h Proj(x) + b_h))
     - Ouput layer f(x) = Softmax(W_o h + b_o)
    Loss ← CrossEntropy(y, f(x))
    Train up to 300 epochs with Adam optimizer
    Enable early stopping (patience = 20) only after epoch ≥ 200
    Save checkpoint with highest validation accuracy
5.  Fine-tuning
    Resume form best checkpoint
    Apply ReduceLROnPlateau scheduler (factor=0.1, patience=5, min_lr=1e-6)
    Fine-tune up to 100 epochs with early stopping (patience = 20)
6.  Evaluation
    Evaluate f(·) on test set and report performance.
```

Table S1. Full list of Drug–Drug Interaction (DDI) types and their definitions. Sample counts are derived from final_interaction.csv.

DDI Type	Description	Number of Samples
1	The risk or severity of adverse effects can be increased when Drug a is combined with Drug b.	60997
2	The metabolism of Drug b can be decreased when combined with Drug a.	34360
3	The serum concentration of Drug b can be increased when it is combined with Drug a.	23779
4	The serum concentration of Drug b can be decreased when it is combined with Drug a.	9506
5	Drug a may increase the hypotensive activities of Drug b.	8397
6	The therapeutic efficacy of Drug b can be decreased when used in combination with Drug a.	7788
7	Drug a may increase the QTc-prolonging activities of Drug b.	6140
8	Drug a may increase the central nervous system depressant (CNS depressant) activities of Drug b.	5435

9	The metabolism of Drug b can be increased when combined with Drug a.	5011
10	Drug a may increase the anticoagulant activities of Drug b.	3160
11	Drug a may decrease the antihypertensive activities of Drug b.	3089
12	Drug a may increase the hypoglycemic activities of Drug b.	2109
13	Drug a may decrease the excretion rate of Drug b which could result in a higher serum level.	1825
14	Drug a may increase the bradycardic activities of Drug b.	1277
15	Drug a may increase the hypokalemic activities of Drug b.	1204
16	Drug a can cause a decrease in the absorption of Drug b resulting in a reduced serum concentration and potentially a decrease in efficacy.	1092
17	Drug a may decrease the cardiotoxic activities of Drug b.	1043
18	Drug a may increase the sedative activities of Drug b.	1015
19	Drug a may increase the neuroexcitatory activities of Drug b.	936
20	Drug a may increase the serotonergic activities of Drug b.	803
21	Drug a may increase the atrioventricular blocking (AV block) activities of Drug b.	716
22	Drug a may increase the hypertensive activities of Drug b.	673
23	Drug a may increase the nephrotoxic activities of Drug b.	664
24	Drug a may increase the antihypertensive activities of Drug b.	629
25	Drug a may increase the orthostatic hypotensive activities of Drug b.	616
26	Drug a may decrease the sedative activities of Drug b.	558
27	The serum concentration of the active metabolites of Drug b can be increased when Drug b is used in combination with Drug a.	538
28	The bioavailability of Drug b can be decreased when combined with Drug a.	519
29	Drug a may decrease the stimulatory activities of Drug b.	498
30	The risk or severity of QTc prolongation can be increased when Drug a is combined with Drug b.	443
31	Drug a may increase the neuromuscular blocking activities of Drug b.	428
32	Drug a may increase the fluid retaining activities of Drug b.	422
33	Drug a may increase the tachycardic activities of Drug b.	372
34	Drug a may decrease the bronchodilatory activities of Drug b.	361
35	Drug a may increase the arrhythmogenic activities of Drug b.	355
36	Drug a may increase the antiplatelet activities of Drug b.	336
37	Drug a may decrease the diuretic activities of Drug b.	324
38	Drug a may increase the anticholinergic activities of Drug b.	323
39	The serum concentration of the active metabolites of Drug b can be reduced when Drug b is used in combination with Drug a resulting in a loss in efficacy.	313
40	Drug a may increase the immunosuppressive activities of Drug b.	312
41	Drug a may decrease the vasoconstricting activities of Drug b.	309
42	Drug a may increase the respiratory depressant activities of Drug b.	300
43	Drug a may increase the analgesic activities of Drug b.	280
44	Drug a may increase the hyperkalemic activities of Drug b.	278
45	The therapeutic efficacy of Drug b can be increased when used in combination with Drug a.	245
46	Drug a may decrease the anticoagulant activities of Drug b.	238
47	Drug a may increase the cardiotoxic activities of Drug b.	202
48	Drug a may increase the hypocalcemic activities of Drug b.	180
49	Drug a may increase the constipating activities of Drug b.	148
50	The risk or severity of bleeding can be increased when Drug a is combined with Drug b.	128
51	Drug a may increase the hyponatremic activities of Drug b.	118
52	Drug a may increase the vasoconstricting activities of Drug b.	109
53	Drug a may increase the thrombogenic activities of Drug b.	108
54	Drug a may increase the antipsychotic activities of Drug b.	94
55	Drug a may increase the adverse neuromuscular activities of Drug b.	94

56	Drug a may decrease the neuromuscular blocking activities of Drug b.	83
57	Drug a may increase the hypercalcemic activities of Drug b.	83
58	Drug a can cause an increase in the absorption of Drug b resulting in an increased serum concentration and potentially a worsening of adverse effects.	82
59	Drug a may increase the myopathic rhabdomyolysis activities of Drug b.	69
60	Drug a may increase the vasopressor activities of Drug b.	65
61	Drug a may increase the hepatotoxic activities of Drug b.	64
62	Drug a may increase the stimulatory activities of Drug b.	56
63	The absorption of Drug b can be decreased when combined with Drug a.	45
64	Drug a may increase the ulcerogenic activities of Drug b.	43
65	Drug a may increase the myelosuppressive activities of Drug b.	34
66	Drug a may decrease effectiveness of Drug b as a diagnostic agent.	33
67	Drug a may increase the vasodilatory activities of Drug b.	33
68	The risk or severity of hypotension can be increased when Drug a is combined with Drug b.	33
69	Drug a may increase the hyperglycemic activities of Drug b.	28
70	Drug a may increase the central nervous system depressant (CNS depressant) and hypertensive activities of Drug b.	27
71	The risk of a hypersensitivity reaction to Drug b is increased when it is combined with Drug a.	27
72	Drug a may increase the bronchoconstrictory activities of Drug b.	26
73	The risk or severity of heart failure can be increased when Drug b is combined with Drug a.	26
74	Drug a may increase the hypotensive and central nervous system depressant (CNS depressant) activities of Drug b.	14
75	The risk or severity of hypertension can be increased when Drug b is combined with Drug a.	14
76	The protein binding of Drug b can be decreased when combined with Drug a.	11
77	The bioavailability of Drug b can be increased when combined with Drug a.	11
78	Drug a may increase the dermatologic adverse activities of Drug b.	11
79	Drug a may decrease the analgesic activities of Drug b.	10

Table S2. Summary of MLP architecture and training settings

Component	Setting
Projection layer	Yes
Number of hidden layers	1
Activation	ReLU
Optimizer	Adam
Loss function	CrossEntropyLoss
Early stopping patience	20

Table S3. Hyperparameter settings for the best-performing feature combinations

Feature Combination			Hidden dimension	Dropout	Learning rate
SSP	PSP	BioBERT			
○	×	×	1792	0.1854	0.000679
×	○	×	1792	0.2391	0.001671

×	×	○	1536	0.1599	0.000621
○	○	×	1024	0.2388	0.000649
×	○	○	1280	0.1744	0.000557
○	×	○	768	0.3524	0.000280
○	○	○	1536	0.1817	0.000173

Table S4. Accuracy by feature combination and MLP layer configuration (ECFP4).

Feature Combination			Projection + Hidden 1	Hidden 1	Hidden 2
SSP	PSP	BioBERT			
○	×	×	0.9495	0.9477	0.9491
×	○	×	0.9292	0.9252	0.9318
×	×	○	0.9584	0.9509	0.9587
○	○	×	0.9566	0.9579	0.9576
×	○	○	0.9581	0.9509	0.9559
○	×	○	**0.9655**	0.9611	0.9630
○	○	○	0.9636	0.9571	0.9554

Table S5. Accuracy by feature combination and MLP layer configuration (ECFP6).

Feature Combination			Projection + Hidden 1	Hidden 1	Hidden 2
SSP	PSP	BioBERT			
○	×	×	0.9581	0.9532	0.9561
○	○	×	0.9589	0.9620	0.9573
○	×	○	0.9637	0.9637	0.9654
○	○	○	0.9619	0.9635	0.9641
